# Calculating the Expected Net Benefit of Sampling for Survival Data: A Tutorial and Case Study

**DOI:** 10.1177/0272989X241279459

**Published:** 2024-09-20

**Authors:** Mathyn Vervaart

**Affiliations:** Department of Health Management and Health Economics, University of Oslo, Oslo, Norway; Clinical Trial Unit, Oslo University Hospital, Oslo, Norway

**Keywords:** expected value of sample information, expected net benefit of sampling, survival data, simulation methods, economic evaluation model, value of information

## Abstract

The net value of reducing decision uncertainty through collecting additional data is quantified by the expected net benefit of sampling (ENBS). The ENBS is the difference between the population-level expected value of sample information (EVSI) for the data collection exercise and the costs associated with the data collection. ENBS calculations for studies that collect survival data are complicated by the need to take into account censoring, and this has limited the application of value-of-information analysis in this setting. In this tutorial article, we present a general-purpose algorithm for calculating the ENBS for a study that collects survival data along with a step-by-step implementation in R. The algorithm is based on recently published methods for simulating survival data and computing EVSI that do not rely on the survival data being from a distribution with any particular parametric form and that can take into account any arbitrary censoring process. We illustrate the method using a real-life case study drawn from an appraisal of pembrolizumab plus axitinib for treating advanced renal cell carcinoma in which the initial decision was informed by immature survival data.

**Highlights:**

- The net value of reducing decision uncertainty by collecting additional data is quantified by the expected net benefit of sampling (ENBS). This tutorial presents a general-purpose algorithm for computing the ENBS for collecting survival data along with a step-by-step implementation in R.
- The algorithm is based on recently published methods for simulating survival data and computing expected value of sample information that do not rely on the survival data to follow any particular parametric distribution and that can take into account any arbitrary censoring process.
- We demonstrate in a case study based on a previous cancer technology appraisal that ENBS calculations are useful not only for designing new studies but also for optimizing reimbursement decisions for new health technologies based on immature evidence from ongoing trials.

## Introduction

In many countries,^1^ health technology assessment (HTA) is used to prioritize the allocation of health care resources. An important component of HTA is health economic evaluation, in which alternative technologies are compared in terms of expected costs and health benefits. Technologies that aim to improve survival require accurate estimates of life expectancy across the relevant patient population. Yet, HTAs are often informed by immature survival data from ongoing trials, in which the clinical event of interest, death, has occurred in only a small proportion of trial participants, particularly for new cancer treatments.^2^ This is a key concern for decision makers who are responsible for deciding on the adoption of new health technologies, such as the National Institute for Health and Care Excellence (NICE) in the United Kingdom.^3^ Between 2011 and 2022, NICE conducted 229 technology appraisals (TAs) of new cancer drugs, of which 158 (69%) relied on immature survival data where death had occurred in less than 50% of the trial participants.^4^ In 85 (37%) cancer TAs, extremely immature survival data with less than 20% of observed death events was used. This can partly be attributed to the introduction of regulatory mechanisms aimed at accelerating assessments and conditional licensing of new pharmaceuticals by the European Medicines Agency^5,6^ and the US Food and Drug Administration^7^ in the last decades.

Estimating long-term survival from immature trial data requires a high degree of extrapolation beyond the observed trial period, necessitating assumptions about disease progression and treatment mechanisms that have not yet been observed. Thus, significant uncertainty about a technology’s long-term effectiveness and cost-effectiveness can remain. This uncertainty increases the risk of recommending a cost-ineffective technology that reduces population net health benefits, by diverting limited resources from more effective interventions and/or by providing patients with an inferior treatment option. Therefore, there may be value in reducing uncertainty by extending an ongoing trial’s follow-up and collecting additional data before making an adoption decision. This is particularly important when decision reversal is expected to be difficult or costly, as past experiences with conditional approval suggest,^8–11^ or when early access to a new technology reduces the probability that additional data will be collected.^12^ In this context, there will be a tradeoff between granting early access to a new technology that may turn out to reduce net health benefits and waiting for uncertainty to be reduced through ongoing data collection with a potential loss of health benefits while waiting.^13,14^

This tradeoff can be quantified by computing the expected value of sample information (EVSI), which measures the expected value to the decision maker of collecting additional data.^15^ EVSI measures the value of collecting additional data by simulating plausible datasets before the actual data collection takes place and then computing the expected costs and health benefits of alternative technologies given both the simulated data and existing evidence. The expected net benefit of sampling (ENBS) is the expected net value of the proposed data collection and can be calculated by subtracting the data collection costs, as well as potential health opportunity costs if approval is withheld while additional data are being collected, from the population EVSI.^16^

Recently, Vervaart et al.^17^ developed efficient general-purpose methods for simulating survival data and for computing the EVSI for collecting survival data from an ongoing trial,^14^ accounting for structural uncertainty about the choice of survival model for extrapolation. There is, however, a lack of clear guidance and practical software for applying ENBS calculations to real-world decision making informed by immature survival data. ENBS calculations for survival data are complicated by the need to take into account censoring, which occurs when the event of interest is not observed for all study participants due to limited follow-up and trial dropout.

In this tutorial, we focus on describing the practical implementation of these efficient EVSI methods and how they can be used to standardize ENBS calculations for collecting additional survival data, using a recent cancer TA by NICE that was informed by immature survival data from an ongoing trial. We provide a step-by-step implementation using R software.^18^ For this implementation, we developed fast and practical functions that automate the ENBS calculations for all possible survival distributions, requiring only the output from a standard probabilistic analysis (PA) as is already the norm in TAs of new pharmaceuticals.^19–22^ The most up-to-date R code to create the analyses in this tutorial can be found in the accompanying GitHub repository (https://github.com/matverv/enbs-survival-tutorial), which can easily be customized to suit specific case studies.

The tutorial is structured as follows. In the second section, we present methods and a general-purpose algorithm for computing the ENBS for collecting additional survival data and introduce R functions that implement this algorithm. In the third section, we introduce the case study and describe the necessary steps and R code to compute the ENBS for the case study. In the fourth section, we discuss the results of the case study. In the final section, we conclude with a brief discussion and provide suggestions for future research.

## Method

### Decision Model

Cost-effectiveness models are commonly used to compare d=1,…,D treatment options (or decisions), such as new treatments and standard care, in terms of expected costs C and health effects H , relative to the decision maker’s willingness-to-pay threshold, λ . The cost-effectiveness of treatment option d can be expressed in terms of net health benefit, $NB_{d}=H_{d}-C_{d}/\lambda$ , or net monetary benefit, $NB_{d}=H_{d}\lambda -C_{d}$ . The optimal treatment is the one that maximizes net benefit. A cost-effectiveness model, which we denote as $NB_{d}\left(\theta \right)$ , predicts the net benefit of treatment option d given a vector of model input parameters, $\theta =\{\theta_{1},\ldots ,\theta_{p}\}$ , such as costs, utilities, and survival probabilities. The input parameters are rarely known with certainty, and plausible ranges for their values are represented in the joint probability distribution p(θ) . The joint distribution p(θ) is usually informed by multiple information sources, such as clinical trials, literature reviews, and expert opinion. The expected net benefits given current information can be estimated in a PA by first sampling values from the distribution of the model parameters, ***θ*** ∗∼ ***p*** (***θ***) and then averaging over the net benefits for each d given the parameter samples, NB_*d*_(***θ*** ∗). The expected net benefit of the optimal treatment option given current information is given by

(1) $$\underset{d}{\max}E_{\theta }\left\{NB_{d}\left(\theta \right)\right\}.$$

### Survival Data

Uncertainty is an inherent aspect of assessing the cost-effectiveness of health technologies, which often arises due to limitations in the available data. This is particularly pronounced for survival data, which are commonly used to inform key decision drivers such as life expectancy and time to disease progression. Survival data describe the time from some well-defined origin, such as treatment initiation, until the occurrence of some particular event or endpoint, such as death or disease progression.^23^ Survival data are often censored due to administrative censoring, which happens when the follow-up time is not sufficient to observe the event of interest for all individuals. Individuals may also be lost to follow-up and drop out of the study due to various reasons, such as relocating to another country or withdrawing consent, leading to another type of censoring known as loss to follow-up. Parametric survival models are therefore often used to extrapolate censored survival data beyond the observed period, typically over the remaining lifetime. To achieve robust extrapolations that accurately predict long-term survival outcomes, it is crucial to have sufficient follow-up data.^24,25^ Yet, survival extrapolations are often informed by immature survival data from trials that are still ongoing, particularly for new cancer drugs.^2,26^ The immaturity of the trial data directly corresponds to the level of uncertainty not only in the survival extrapolation but also, consequently, in the cost-effectiveness estimates.^24,26^ Uncertainty stemming from immature survival data can therefore result in a greater risk of recommending a cost-ineffective technology that reduces population net health benefits.

The expected value of eliminating uncertainty about the survival parameters can be quantified by computing the expected value of partial perfect information (EVPPI).^27^ However, obtaining perfect information is rarely possible. Instead, we may reduce uncertainty about the survival parameters by delaying a decision to approve or reject a new health technology from current time $t_{1}$ to future point $t_{2}$ , so that additional survival data, which we denote as X , can be collected in the ongoing trial.^14^ The value of X is derived from its potential to change the optimal decision; that is, new data may reveal that a technology that was previously considered cost-effective turns out to be cost-ineffective. Optimal decision making therefore requires determining the point at which follow-up can be considered sufficiently mature.

### Expected Value of Sample Information

At the point of decision making, X is yet uncollected, and we therefore compute the expected value of X by taking the expectation over the distribution of all possible realizations of the data at future follow-up time $t_{2}$ . This usually means we need to simulate a large number of plausible datasets from the distribution of the data, x~p(X) . We can simulate datasets by first sampling from the joint distribution of the model parameters, ***θ*** ∗∼*p* **(*θ*)**, and then simulating from the sampling distribution of the data given the parameter sample, x ***∗∼ p*** (***X*** | ***θ*** ∗). This generates a pair of samples {x∗,∗} from the joint distribution p(X,θ) , and therefore, the samples $x^{*}$ are draws from the marginal distribution p(X) .^28^

The EVSI of extending the follow-up from current time $t_{1}$ to future point $t_{2}$ is equal to the average gain in net benefit over all possible future datasets,^29^

(2) $$\mathrm{EVSI}=E_{X}[\underset{d}{\max}E_{\theta |X}\{NB_{d}\left(\theta \right)\}]-\underset{d}{\max}E_{\theta }\{NB_{d}\left(\theta \right)\}.$$

where the first term is the expected net benefit of a decision made with new data, X , and the second term is the expected net benefit of a decision made with current information.

### Computing EVSI for Survival Data

To compute the EVSI, we need to perform three main steps:

1. Sample from the joint distribution of the model parameters and compute the expected net benefits given current information in a PA.
2. Simulate new study datasets conditional on the PA sample.
3. Compute the posterior expected net benefits given both the simulated datasets and current information.

#### Probabilistic analysis

To compute the expected net benefits given current information, we first sample k=1,…,K values, ***θ***^(*k*)^, from the joint distribution of the cost-effectiveness model parameters. A subset of ***θ***^(*k*)^ often includes survival model parameters that are used to construct survival curves, $s^{\left(k\right)}=\{s_{0}^{\left(k\right)},\ldots ,s_{T}^{\left(k\right)}\}$ , which are vectors of survival probabilities $s_{t}^{\left(k\right)}$ at fixed cycle times t=0,…,T , as distinct from S(t) , which is commonly used to denote the continuous-time survivor function. From $s^{\left(k\right)}$ , the distribution of patients across the health states over time can be derived, such as the proportions of patients who are progression-free or have progressed. The choice of survival model can heavily influence long-term survival estimates and requires careful consideration of factors such as observed and expected trends in the hazard of death and the plausibility of long-term survival estimates. Importantly, there is often uncertainty about the most appropriate survival model to extrapolate the observed data, and this uncertainty could be formally accounted for using model averaging.^30^ We refer the reader to the literature for further guidance on survival model selection.^31,32^

The total costs $C_{d}^{\left(k\right)}$ and quality-adjusted life-years $H_{d}^{\left(k\right)}$ for each treatment option d can be computed by multiplying the distribution of patients across the health states with their respective health state costs and health-related quality-of-life weights. We then calculate the net benefits for each treatment option given threshold value λ , ${\mathrm{NB}}_{d}^{\left(k\right)}=H_{d}^{\left(k\right)}-C_{d}^{\left(k\right)}/\lambda$ .

#### Simulating survival datasets

Vervaart et al.^17^ proposed 2 general-purpose methods, a discrete sampling method and an interpolated inverse transform sampling (ITS) method, for simulating survival data $x^{\left(k\right)}$ from a probabilistic sample of survival probabilities over discrete cycle times $s^{\left(k\right)}$ . The discrete sampling method achieves this by sampling the model cycles {1,…,T} using the increments between the sampled survival probabilities $s^{\left(k\right)}$ and setting the event times to the half-cycle times. The interpolated ITS method extends this to continuous time by initially sampling random uniform numbers between 0 and 1 and then interpolating the survival probabilities using monotone cubic splines at the sampled numbers and recording the interpolated cycle times. These general-purpose methods greatly reduce the computational burden of the EVSI data-simulation step when we assume treatment effect waning or use flexible survival models, such as relative survival models, spline models, and mixture cure models,^32^ as they do not require numerically evaluating the integrals and function inverses for these more complex survival distributions.^17^ Furthermore, the implementation of the general-purpose data-simulation methods is identical across all possible survival distributions and can therefore easily be standardized from a traditional PA.

It is important to account for both administrative censoring and censoring due to loss to follow-up when simulating survival times. We can apply administrative censoring by specifying the time at which the study ends or the next interim analysis takes place and then taking the minimum of the simulated survival times and the time point at which administrative censoring will take place. The probability of being lost to follow-up can be modeled using an independent parametric survival model if loss to follow-up is assumed to be independent of the event of interest or a joint model if there is dependency between censored and observed events. We record the survival time corresponding to the earliest occurrence of the 3 possible events: the event time, the administrative censoring time, and the censoring time due to loss to follow-up. We also define a censoring indicator to denote whether the recorded survival time corresponds to an observed event or a censoring event.

Mathematically, a single simulated dataset, $x^{\left(k\right)}$ , consists of i=1,…,n survival times $x_{i}^{\left(k\right)}$ and censoring indicators $\delta_{i}^{\left(k\right)}$ , $x^{\left(k\right)}=\{x_{1}^{\left(k\right)},\ldots ,x_{n}^{\left(k\right)},\delta_{1}^{\left(k\right)},\ldots ,\delta_{n}^{\left(k\right)}\}$ , where $\delta_{i}^{\left(k\right)}=1$ when $x_{i}^{\left(k\right)}$ is an observed event, and $\delta_{i}^{\left(k\right)}=0$ when $x_{i}^{\left(k\right)}$ is a censored observation.

#### Computing posterior net benefits

A practical choice for computing the posterior net benefits conditional on the simulated datasets is a fast nonparametric regression-based method,^14^ which does not require a parametric distribution for the simulated data. The nonparametric regression-based method estimates the posterior net benefits by regressing the net benefits on a summary statistic of the simulated datasets.

The regression-based method works as follows. First, for each treatment option d , we need to compute a low-dimensional summary statistic for the simulated datasets $x_{d}^{\left(k\right)}$ , denoted $T(x_{d}^{\left(k\right)})$ . Typically, as suggested by Strong et al.,^33^ this would involve computing the sample estimates of the model parameters that are updated by the new data and selecting these estimates as the summary statistic. However, an alternative summary statistic has been proposed by Vervaart et al.^14^ in the context of survival data; the number of observed events $e_{d}^{\left(k\right)}=\sum_{i=1}^{n}\delta_{\mathrm{di}}^{\left(k\right)}$ and the total time at risk $v_{d}^{\left(k\right)}=\sum_{i=1}^{n}x_{\mathrm{di}}^{\left(k\right)}$ , thus $T(x_{d}^{\left(k\right)})=\{e_{d}^{\left(k\right)},v_{d}^{\left(k\right)}\}$ . This generic summary statistic does not depend on the survival data being from a distribution with any particular parametric form, is straightforward to calculate, and has been shown to give accurate results for various survival models and hazard shapes.^14^ To reduce the number of regression equations from d to d−1 , we compute the incremental net benefit (INB) for each d , ${\mathrm{INB}}_{d}^{\left(k\right)}={\mathrm{NB}}_{d}^{\left(k\right)}-{\mathrm{NB}}_{1}^{\left(k\right)}$ , where ${\mathrm{NB}}_{1}^{\left(k\right)}$ are usually the net benefits for standard care ( d=1 ). Note that the results are not sensitive to the choice of common comparator when calculating the INB. We then regress the INBs on the summary statistics of the simulated data using a flexible nonparametric regression model, which we denote as g .

(3) $$\mathrm{IN}B_{d}(\theta^{\left(k\right)})=g\{T(x_{d}^{\left(k\right)})\}+\varepsilon ,$$

where ε is a zero-mean error term.

We then extract the regression model–fitted values ${\hat{g}}_{d}^{\left(k\right)}$ , which are estimates of the posterior INBs conditional on the simulated datasets for each d . The regression-based estimate of the EVSI in equation (2) is given by

(4) $$\mathrm{EVSI}\approx \frac{1}{K}\sum_{k=1}^{K}\underset{d}{\max}{\hat{g}}_{d}^{\left(k\right)}-\underset{d}{\max}\frac{1}{K}\sum_{k=1}^{K}{\hat{g}}_{d}^{\left(k\right)}.$$

##### Population-level EVSI

To establish the population-level EVSI, an assessment of the number of current and future patients who may benefit from additional data collection over the decision relevance horizon is required.^34^ The total beneficiary population, P , can be estimated from the number of patients who would be affected by the treatment decision in a given year, $P_{z}$ , which consists of both incident patients as well as a potential “catch-up” cohort of prevalent patients. The appropriate decision relevance horizon in years, which we denote as Z , depends on uncertain events in the near future, such as large price reductions and the launch of new technologies, and should be seen as a proxy for the complex and uncertain process of future change.^34^ Information about a new treatment can hold value beyond the current decision, as newly introduced treatments often become the comparator in future economic evaluations. In practice, values ranging from 5 to 15 y have been used,^35^ although this may vary depending on the disease area and technology being studied and should be subjected to a sensitivity analysis. Rare diseases, for example, tend to have a less dynamic treatment landscape with fewer new treatments entering the market compared with other disease areas, such as oncology.^36^ Furthermore, as the follow-up time increases, the number of patients who will be able to benefit from the additional research decreases. This should be accounted for by reducing the value of Z for the proposed follow-up duration. It is also important to account for the lag time between the end of follow-up and the implementation of an adoption decision in clinical practice, which includes data analysis, reporting, the appraisal process, price negotiations, and updating of guidelines, as this reduces the remaining decision relevance horizon.

We can compute the total beneficiary population as

(5) $$\begin{array}{c} P=\sum_{z=0}^{Z}\frac{P_{z}}{{\left(1+r\right)}^{z}},\; \end{array}$$

where r is the annual discount rate used in the health economic model.^35^

While a positive population-level EVSI quantifies the expected value of the new data, we also need to consider the costs of collecting the data to determine whether additional data collection is worthwhile.

#### Expected net benefit of sampling

The ENBS quantifies the expected net value of additional data collection.^37^ The ENBS is the difference between the population-level EVSI for collecting new data and the expected cost of continuing an ongoing study and/or conducting a new study and any potential health benefits that may be foregone if approval is withheld. A positive ENBS indicates that it is worthwhile to continue or conduct the study and collect more data before making an adoption decision.

##### Costs of data collection

The costs of conducting a new study include fixed costs that remain constant regardless of the sample size or length of follow-up, such as protocol development, approvals, site setup, database development costs, and analysis costs. In addition, variable trial costs, such as recruitment, follow-up, site and database management, and analysis costs, can depend on both the sample size and the length of follow-up.^38^ Costs of continuing an ongoing study primarily consist of variable costs, as fixed trial costs have already been incurred. Study costs are often specified as research costs in funding applications and can be defined in collaboration with study experts.^35^

In determining relevant costs, it is important to consider the perspective of the decision maker. For instance, the costs of completing an ongoing study for a new technology have already been committed by the manufacturer and are not borne by society. If patient access to a new treatment is delayed until the data have been collected, there is an opportunity cost to society in terms of potential forgone health benefits that could have been avoided by providing a cost-effective treatment during the data collection period. This opportunity cost can be quantified as the expected INB generated by the new treatment compared with standard care.

##### Decision options for additional data collection

There are 2 decision options when additional data collection is considered, in addition to approving or rejecting a new technology: approval with research (AWR) or only in research (OIR). AWR refers to approval while additional data are being collected, whereas OIR means a decision to approve or reject is withheld until additional data have been collected.^12^ OIR may be more appropriate if there are significant irrecoverable costs, such as high upfront treatment or capital investment costs, that could be avoided if treatment initiation could be delayed until additional data have been collected, such as for chronic diseases, or if the new technology is not expected to improve net health benefits but there is still value in collecting additional data to reduce uncertainty.^39^ OIR may also be the only option if the adoption decision is difficult or costly to reverse, including the time and effort required to change clinical guidelines and practice and switch patients over to an alternative treatment or when approval would adversely affect the prospects of further data collection. In these circumstances, there will be opportunity costs in terms of potential net health benefits foregone as patients receive a suboptimal treatment during the data collection period.

A generic algorithm for computing the ENBS for collecting survival data in a new or ongoing study using the previously described general-purpose data-simulation methods^17^ and regression-based EVSI method^14^ is given in Box 1.

**Box 1:** Generic ENBS Algorithm for Collecting Survival Data

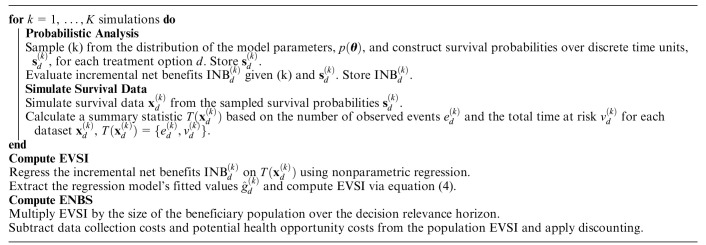

ENBS, expected net benefit of sampling; EVSI, expected value of sample information; INB, incremental net benefit.

## Case Study: Pembrolizumab plus Axitinib for Treating Renal Cell Carcinoma

We based our case study on a recent single TA by NICE of pembrolizumab plus axitinib for treating advanced renal cell carcinoma, named TA650.^40^ Key clinical evidence in TA650 was obtained in an interim analysis of the KEYNOTE-426 trial, an open-label phase 3 trial comparing pembrolizumab plus axitinib with sunitinib in patients with previously untreated advanced clear-cell renal-cell carcinoma.^41^ Overall survival (OS) data were immature at the time of the appraisal, as only 156 (18%) of 861 enrolled patients had died during a median follow-up of 12.8 mo. According to the trial protocol, follow-up for OS would continue until 404 (47%) deaths had been observed, after which the study would be concluded.^42^

### Health Economic Model

We reconstructed the cost-effectiveness model that was used in TA650 using publicly available information from the NICE committee papers.^40^ The model was based on a partitioned survival model (PSM) structure with 3 health states: progression free, progressive disease, and death. We used the algorithm by Liu et al.^43^ to reconstruct individual patient data (IPD) for OS and progression-free survival (PFS) from published Kaplan-Meier (KM) curves for the KEYNOTE-426 trial.^41^ This algorithm uses vector images to extract the coordinates of the KM curves, thereby improving accuracy and minimizing observer variation compared with alternative IPD reconstruction methods that rely on manual digitization.^44,45^ Plots of the reconstructed KM data can be found in Appendix A, while the R code for the IPD reconstruction is available in the accompanying GitHub repository. The proportion of patients in the health states over time were derived directly from the OS and PFS curves fitted to the reconstructed IPD.^46^ The PSM estimated the expected costs, life-years, and quality-adjusted life-years (QALYs) for pembrolizumab plus axitinib and for sunitinib at a discount rate of 3.5% a year, given a time horizon of 40 y and weekly model cycles. Definitions and prior distributions for the model input parameters are given in Appendix B. We first validated the reconstructed model by comparing the predicted costs, QALYs, and life-years with the company’s base-case estimates in TA650 (Appendix C). We then implemented a number of assumptions that were preferred by the NICE appraisal committee, of which the most notable were the consideration of both a loglogistic and exponential model for OS for pembrolizumab plus axitinib and waning of the treatment effect by year 5 following a 2-y treatment-stopping rule for pembrolizumab. We captured uncertainty about choosing between a loglogistic and exponential model using model averaging with model weights defined by the Akaike’s information criterion.^30^ We implemented treatment effect waning using a previously described additive hazard approach^17^ and sampled values for the start and duration of the waning period from lognormal distributions with means of 2 and 3 y, respectively. We then computed vectors of “waning hazards” from the mean survival probabilities for pembrozilumab plus axitinib and sunitinib that in linear proportions increase from no waning to full waning over the sampled waning periods. We then added the waning hazards to each of the sampled OS and PFS hazards for pembrozilumab plus axitinib, resulting in hazards that in expectation converge to the expected hazards in the sunitinib arm, while for each iteration in the PA preserving independence between survival endpoints and ensuring that treatment effect waning can only reduce the treatment effect. Drug prices are often set to ensure an incremental cost-effectiveness ratio slightly below the willingness-to-pay threshold, a strategy known as “value-based pricing.” In our analysis, given that pembrolizumab plus axitinib was not cost-effective at the list price, we assumed such a value-based price to reflect this common scenario (Appendix B).

### Analysis

The following section features R code snippets. Unless specified otherwise, we are using base R for the analysis. The comprehensive R code used in this tutorial can be found in the corresponding GitHub repository, available at https://github.com/matverv/enbs-survival-tutorial. The implementation of the algorithm requires no advanced statistical expertise or specialized programming. Furthermore, direct access to the full cost-effectiveness model is not necessary, as the algorithm requires only the output from the PA. While the code in this tutorial focuses on OS due to the context of the case study, it can easily be adapted to incorporate any number of treatments and survival outcomes, including PFS. An example demonstrating the calculation of EVPPI, EVSI, and ENBS for both OS and PFS, along with an additional example for a new trial, can be found in the accompanying GitHub repository. We follow the naming conventions introduced by Alarid-Escudero et al.^47^ and use the prefix l for lists, df for data frames, m for matrices, and v for vectors.

#### Probabilistic analysis

We begin our analysis by conducting a PA with K=5,000 simulations by invoking the run_pa_pembro function. This function runs a PA using the reconstructed cost-effectiveness model used in TA650 and provides the following outputs:

- m_c: A matrix of costs, where each row represents a simulation, and each column corresponds to a treatment strategy. The first column corresponds to treatment with sunitinib ( d = 1) and the second column to treatment with pembrolizumab plus axitinib ( d = 2).
- m_e: A matrix of QALYs, formatted similarly to the m_c matrix.
- l_surv: A list that stores matrices of survival probabilities for any number of treatments and survival outcomes, where rows index the model cycles and columns index the PA simulations. In our case study, it specifically contains 4 matrices: 2 for OS probabilities (m_os1 and m_os2) and 2 for PFS probabilities (m_pfs1 and m_pfs2), corresponding to each treatment strategy.

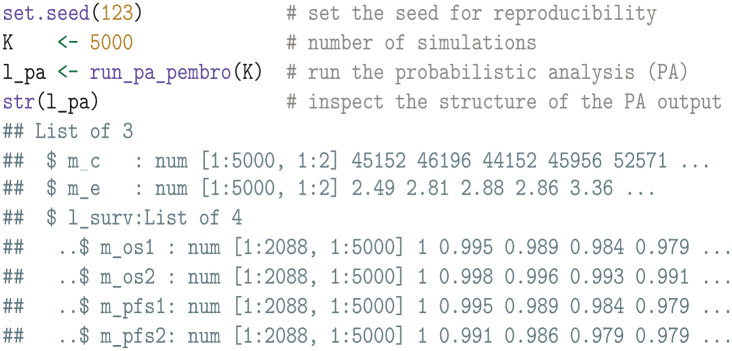


A cost-effectiveness scatterplot, as well as a plot of the simulated OS and PFS curves, can be found in Appendix D in the supplemental material. Note that the run_pa_pembro is tailored specifically for this case study and is not described in further detail in this tutorial. For other studies, analysts must develop their own cost-effectiveness model and PA function, ensuring that the results conform to the format demonstrated above. However, the remaining functions introduced in this tutorial are generic and applicable to a wide range of case studies.

Additional instructions for analysts who conduct their cost-effectiveness analysis and PA in Excel are given in the README file of the accompanying GitHub repository.

We then calculate the net health benefits given a willingness-to-pay threshold of £30,000 per QALY gained.


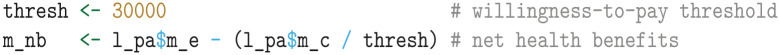


##### Expected value of partial perfect information

The EVPPI for any set of treatments and survival outcomes can be calculated by regressing the net health benefits on the mean survival estimates using a flexible nonparametric regression model. We perform this computation using the evppi function in the voi package, which is available from the Comprehensive R Archive Network (CRAN).^48^ The regression method defaults to a generalized additive model for up to 4 parameters and Gaussian process regression for 5 or more parameters.^49^ Since follow-up in the KEYNOTE-426 trial was still ongoing for OS but had concluded for PFS, our analysis will focus on OS.


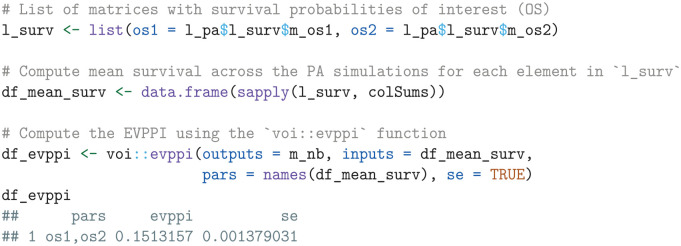


The resulting output df_evppi contains columns for the parameters (pars), the corresponding EVPPI estimate (evppi), and the standard error (se). If additional parameters such as costs and quality of life are of interest, the simulated parameter values can simply be combined with the df_mean_surv data frame prior to the EVPPI calculation.

#### Survival data simulation

##### Data-simulation settings

To compute the EVSI for additional follow-up for OS in the KEYNOTE-426 trial, we must simulate plausible survival times for patients at risk at current follow-up time $t_{1}$ . The simulated survival times must be conditioned on patients having already survived until $t_{1}$ , which requires left-truncating the distribution of the data at $t_{1}$ .

We start by loading the reconstructed IPD containing vectors of survival times (times), event indicators (event), and treatment assignments (treat).


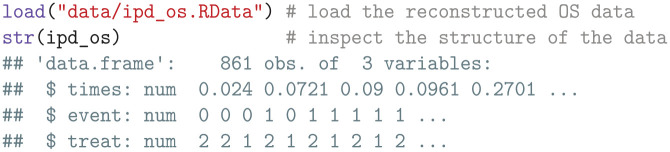


We then subset the reconstructed IPD to identify the censoring times for patients at risk at $t_{1}$ for each treatment arm.


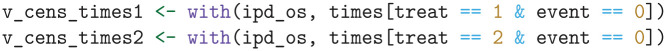


However, we have now selected all censoring times, while we require only the administrative censoring times for the patients who are still enrolled in the trial at $t_{1}$ . We therefore need to exclude the patients who dropped out of the trial from the vectors of censoring times. The number of patients who withdrew from the trial or were noncompliant was 20 for sunitinib and 15 for pembrolizumab plus axitinib.^41^ Since we do not know which of the censoring times corresponds to the patients who dropped out of the trial, we assume these are distributed at evenly spaced intervals among the censoring times. To account for this, we create a remove_dropouts function, which subtracts the trial dropouts from the censoring times at evenly spaced intervals. The resulting administrative censoring times are then stored in a list.


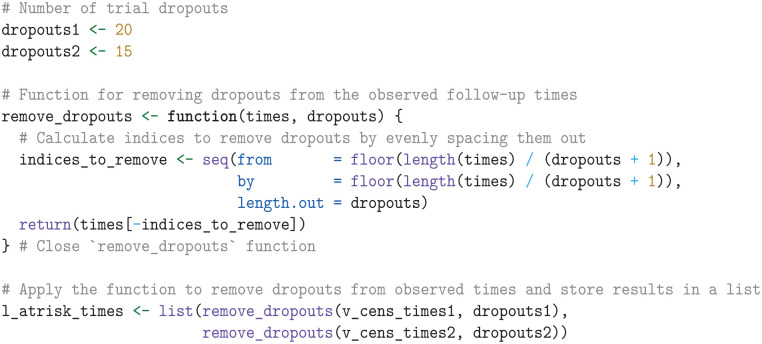


If (reconstructed) IPD is unavailable, we can approximate the censoring times for patients at risk in each arm using rep(med_fu, n), where med_fu is the median follow-up and n represents the number of patients per arm.

We also need to account for the possibility of future dropouts in the EVSI data-simulation step. To do this, we specify shape and rate parameters for the Gamma distribution of the trial dropout rate, informed by the observed dropouts and total time at risk in months for each treatment arm. These are intended for later use by the data-generation function, where they will be used to sample dropout rates.


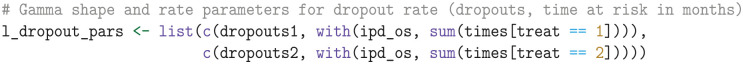


If dropout rates are unknown, we can refer to similar studies or assume negligible dropout by setting the shape and rate close to zero and one, for example, c(0.01, 1), respectively, for each treatment and outcome in the l_dropout_pars list. Although this assumption may overestimate the effective sample size and EVSI, its impact is likely minimal when dropout is expected to be low and could be assessed through sensitivity analysis.

We then define 4 additional administrative censoring times, specified in months and ranging from 3 to 60 mo, evenly spaced on the log scale. These administrative censoring times will be used in the data simulation step and subsequently for calculating and interpolating the EVSI.


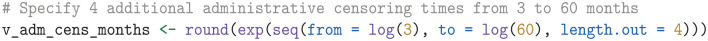


##### Data simulation

Now that we have established the data-simulation settings, we define the function sim_surv_data_ongoing to simulate survival data for an ongoing trial. This function uses the interpolated ITS method^17^ to simulate survival times for a single treatment and survival outcome, applies administrative and dropout censoring, and outputs the observed events and time at risk for each simulated dataset.


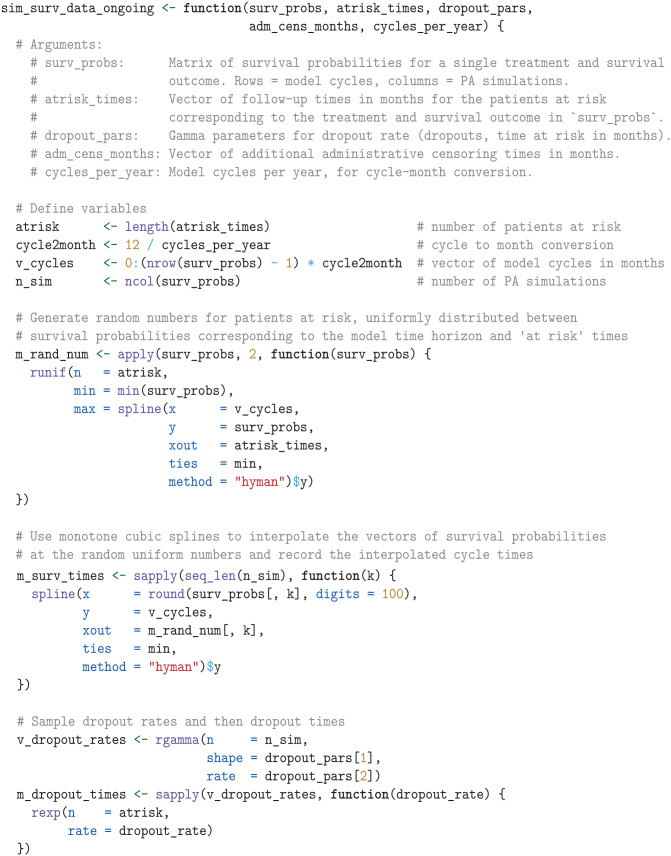



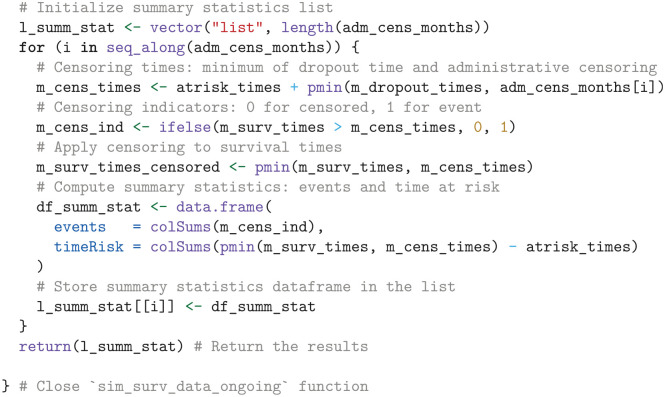


We then use the sim_surv_data_ongoing function to simulate OS data for sunitinib and pembrolizumab plus axitinib. We use the Map function to iterate through corresponding lists of survival probabilities (l_surv), at-risk times (l_atrisk_times), and dropout parameters (l_dropout_pars) and consolidate the resulting output for all treatments and outcomes into the list l_summ_stat. Each element in l_summ_stat is a data frame that contains the simulated observed events and times at risk for a specific administrative censoring time.


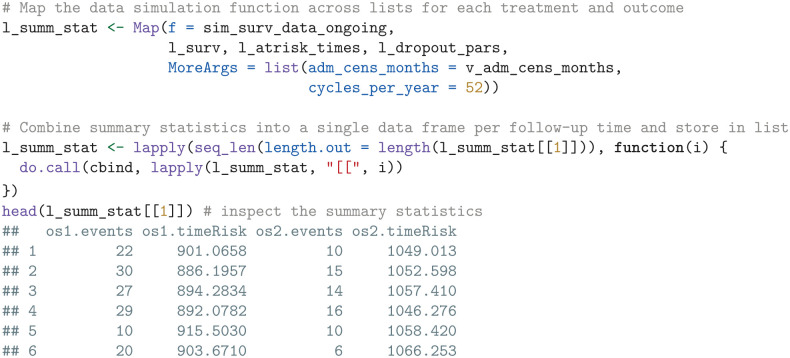


If additional parameters such as costs and quality of life are of interest for the EVSI calculations, define corresponding data-simulation functions to simulate data and calculate appropriate summary statistics. These summary statistics can then be combined with each data frame in the l_summ_stat list. For guidance on simulating data for other outcome types, see Heath et al.^28^

We estimate the additional follow-up time required to reach the trial protocol’s specified end of follow-up through spline interpolation of the simulated number of events.


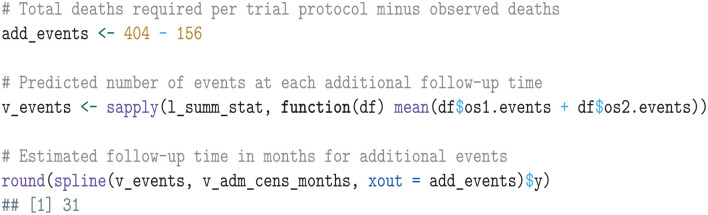


In the next step, we use the simulated summary statistics to compute the EVSI.

#### Expected value of sample information

We can compute the EVSI by regressing the net health benefits on the summary statistics based on the number of observed events and time at risk. To achieve this, we define a function that computes the EVSI using the regression methods in the voi package’s evppi function.


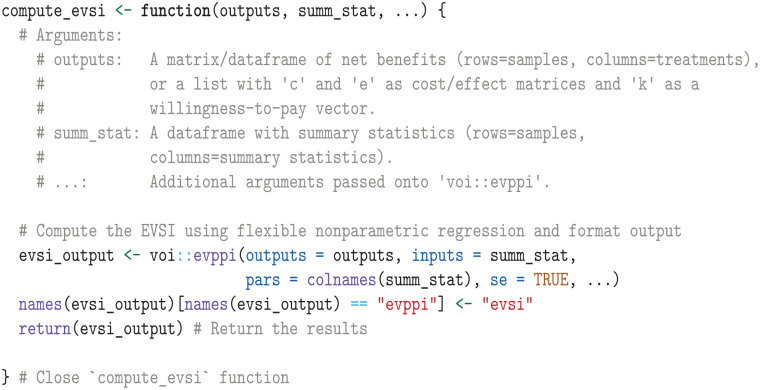


We then apply the compute_evsi function by iterating through the list of summary statistic data frames (one for each administrative censoring time) and format the output.


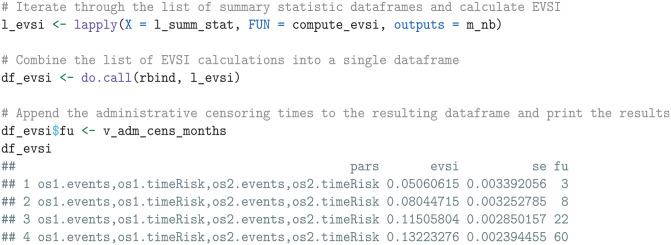


The resulting output df_evsi contains columns for the parameters (pars), the EVSI estimates (evsi), the standard errors (se), and the additional follow-up times (fu).

Next, we define a function that interpolates the EVSI estimates and standard errors over monthly increments. For interpolating the EVSI, we use an asymptotic regression model implemented in the AR.3 function in the drc package, available on CRAN.^50^ For interpolating the log-transformed standard errors, we use smoothing splines in base R.


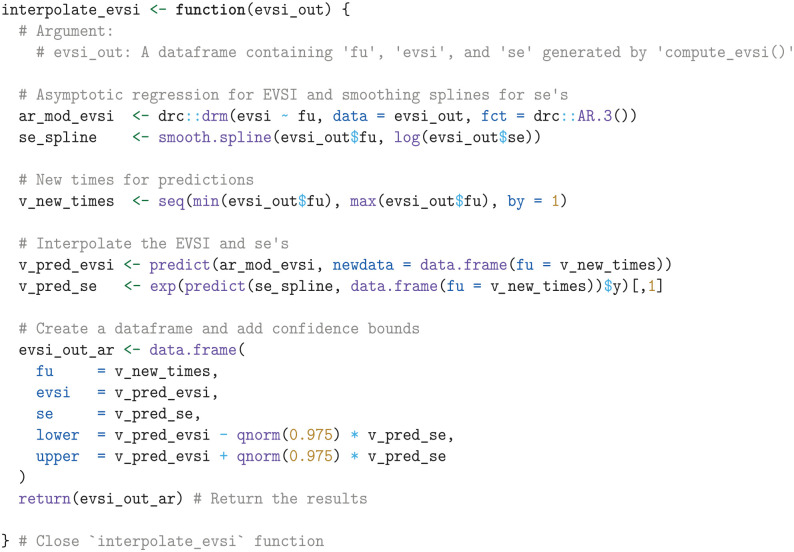


**Figure 1.**
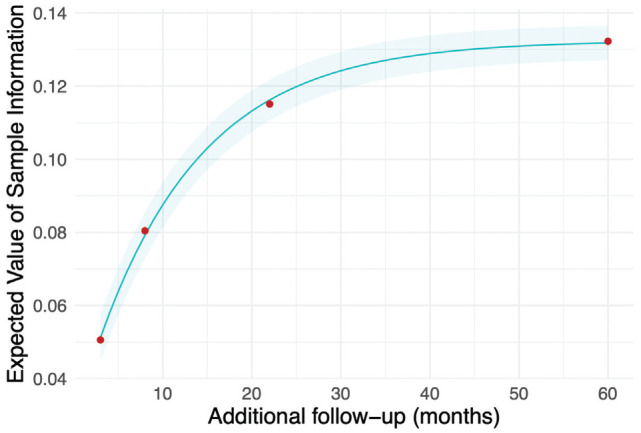
Expected value of sample information for additional follow-up for overall survival in KEYNOTE-426, starting from the point of the first interim analysis.

We then apply the interpolate_evsi function and plot both the interpolated EVSI along with its confidence intervals and the raw EVSI estimates, using the ggplot2 package from CRAN (Figure 1).^51^


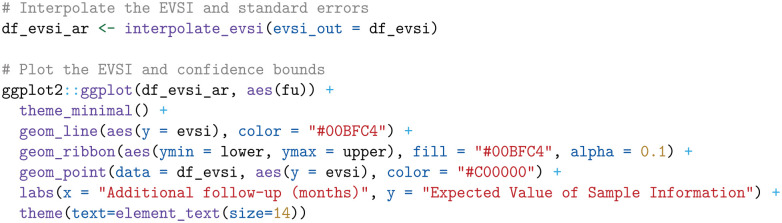


#### Expected net benefit of sampling

To compute the ENBS for AWR and OIR, we define a function named compute_enbs. This function takes several arguments, including the interpolated EVSI estimates, net benefits, fixed and variable trial costs, time lags for AWR and OIR, incidence and prevalent patient populations, the decision relevance horizon, the probability that a decision can be reversed if AWR is chosen, and an annual discount rate. The function calculates the ENBS for both AWR and OIR options and returns a data frame containing the ENBS values across a range of follow-up times.


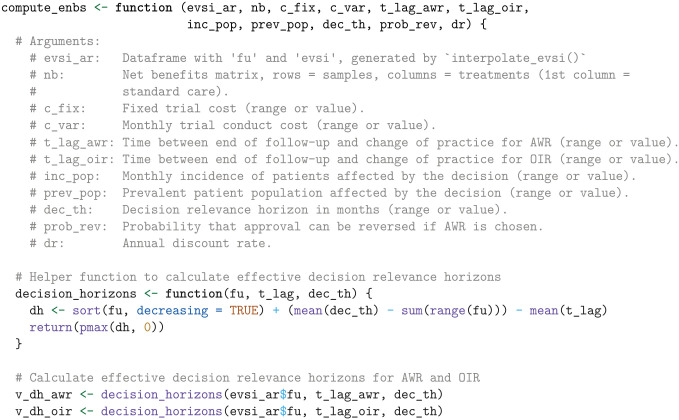



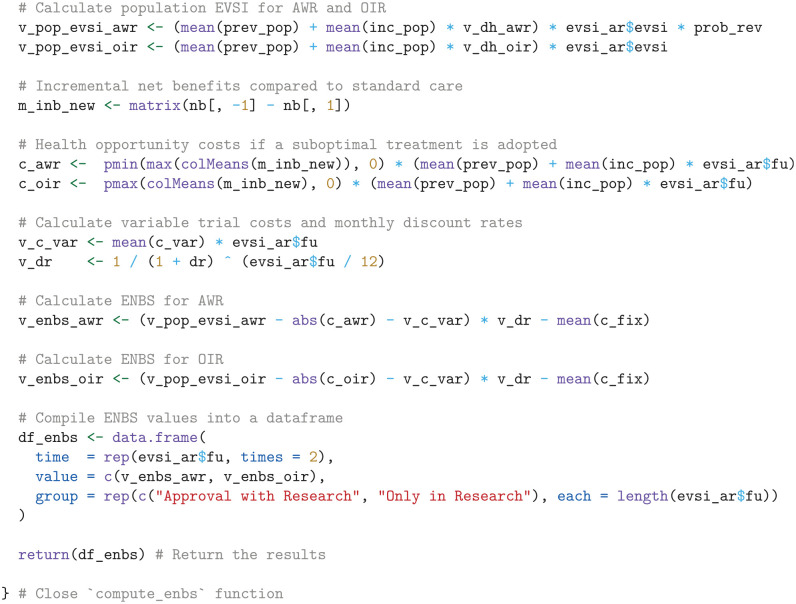


The ENBS computation relies on several assumptions:

- We assume a fixed cost of £100,000 for the NICE reappraisal.
- We assume the National Health Service (NHS) incurs no additional costs for the KEYNOTE trial’s continued OS follow-up, as the manufacturer bears this expense per trial protocol.
- We assume a lag time of 12 mo and 9 mo for AWR and OIR, respectively. The lag time is the duration from the end of follow-up at $t_{2}$ until the change of clinical practice following a reappraisal decision. AWR involves a longer lag time to account for additional delays such as switching patients back to standard care and increased difficulty in negotiating prices. The initial NICE TA, which was published on September 30, 2020, was informed by follow-up data with a cutoff on January 6, 2020. Therefore, the initial NICE appraisal relied on trial data that was approximately 9 mo old at the time of publication. To avoid overestimating the lag time at $t_{2}$ , we reduce the assumed lag by subtracting the time between the current follow-up at $t_{1}$ and the initial TA from the AWR and OIR lag times. This adjustment acknowledges that the decision relevance horizon starts from the initial TA, highlighting that follow-up has already advanced beyond $t_{1}$ at that point.
- From the company submission,^40^ we derived a monthly incidence of 277 patients with advanced renal cell carcinoma in the United Kingdom.
- For illustrative purposes, we assume that 50 prevalent patients are eligible for the new treatment.
- We use an annual discount rate of 0.035 and assume a decision relevance horizon of 90 mo.
- Finally, under AWR, we consider a 100% probability of reversing an approval decision, albeit with an additional delay specified by lag_awr.

Using these inputs and assumptions, the ENBS is computed as follows:


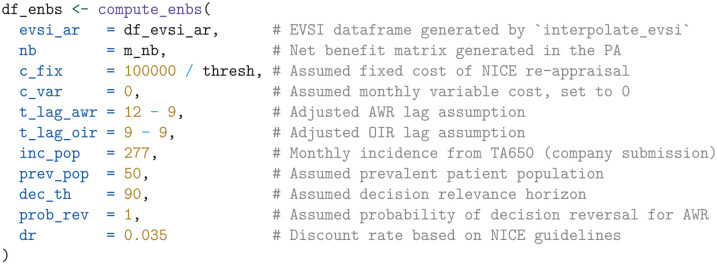


Next, we visualize the ENBS values over the range of follow-up times, highlighting the optimal follow-up times that maximize the ENBS for both AWR and OIR, using the ggplot2 package.


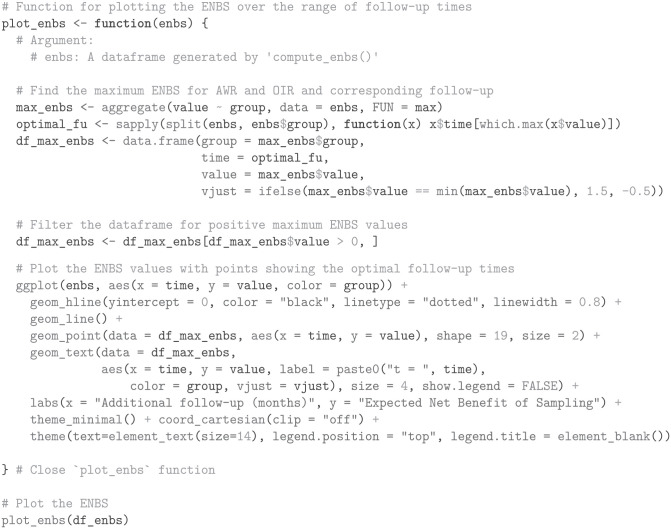


### ENBS Analysis

**Figure 2.**
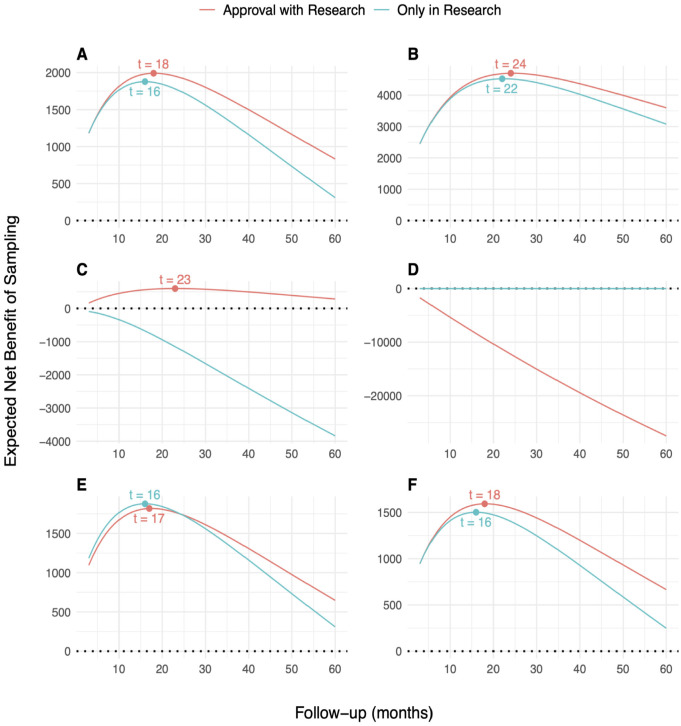
Expected net benefit of sampling (ENBS) for additional follow-up for overall survival in KEYNOTE-426 for the base-case analysis (a) and alternative scenarios (b–f). AWR, approval with research; OIR, only in research. (a) Base case, (b) decision relevance horizon of 180 mo, (c) 50% price discount,^a^ (d) Threshold set at a health opportunity cost of £8,000 per QALY gained,^b^ (e) additional reversal delay of 6 mo for AWR, and (f) 20% lower incidence. ^a^Price discount is relative to the assumed value-based price for pembrolizumab. ^b^Obtained from Martin et al.^52^

Figure 2 presents the ENBS for additional OS follow-up in KEYNOTE-426 for the base-case and alternative scenarios. In the base-case analysis (Figure 2a), the ENBS initially increases as the EVSI increases and then decreases as the population who can benefit from the additional research declines with longer follow-up. The positive ENBS values for both AWR and OIR indicate that there is net value in collecting additional survival data with longer follow-up before making a final reimbursement decision. AWR has a greater ENBS and is therefore a better option than OIR across the range of follow-up times. The optimal decision option that maximizes the ENBS is AWR with an additional follow-up of 18 mo. This indicates that if the adoption decision can be fully reversed, and assuming value-based pricing, pembrolizumab plus axinitib should be conditionally approved and reappraised after 18 mo of additional follow-up for OS. These additional follow-up periods do not require an extension of the planned follow-up period per trial protocol, which is expected to conclude after 31 mo of additional data collection. The maximum ENBS for OIR is greater than that of AWR in 2 scenarios: when the threshold is set equal to the highest most recent estimate of the marginal cost per QALY in the NHS of £8,000^52^ (Figure 2d), in which case pembrolizumab plus axitinib cannot be considered cost-effective and AWR is not an option, and when assuming an additional decision reversal delay of 6 mo for AWR (Figure 2e). Figure 2c illustrates that if the price for pembrolizumab is substantially reduced, the ENBS for OIR becomes negative, and pembrozilumab plus axitinib should be conditionally approved, even if approval cannot be reversed at a later point. The ENBS for AWR and OIR increases with a longer decision relevance time horizon (Figure 2b), whereas a lower incidence rate leads to a reduction in ENBS for both decision options without affecting the optimal follow-up period (Figure 2f).

## Discussion

### Strengths and Limitations

We have presented a tutorial for computing the ENBS for collecting survival data and a step-by-step implementation using R software.^18^ We have developed practical R functions that standardize the key calculations by using recently proposed general-purpose data-simulation^17^ and EVSI methods.^14^ Our functions account for important considerations such as health opportunity costs, administrative censoring and censoring due to trial dropout, lag time between the end of follow-up and change of clinical practice, the decision relevance horizon, the incident and prevalent patient population, costs of setting up and conducting a trial, as well as trial enrollment rates (with additional details available in our online GitHub Appendix). Moreover, our methods account for structural uncertainty, such as uncertainty about the choice of survival model or the point at which to start the extrapolation, as long as the analyst is able to derive probability weights for the competing scenarios and include these in the PA.^14,53^ We demonstrated in a case study based on a previous cancer TA at NICE^40^ that our functions can easily be used to automate ENBS calculations for immature survival data from a standard PA, irrespective of the assumed survival distributions.

In the United Kingdom, many new oncology drugs with an uncertain evidence base are conditionally approved by NICE through the Cancer Drug Fund (CDF) to provide early patient access while additional data are being collected.^54^ Evidential uncertainty often arises due to immature survival data, which has largely been addressed by collecting data with longer follow-up from ongoing trials.^26,55^ Trial data with longer follow-up then form the basis for a review appraisal to inform a final decision on routine commissioning, which should generally take place within 2 y after a drug enters the CDF. However, without a formal assessment of the benefits and costs of the additional data collection, the data collection period may be too long or too short and does not take into account that approval decisions can be difficult or costly to reverse, particularly on the basis of cost-effectiveness arguments, in which case OIR may be more appropriate. For instance, experiences with conditional approval for new pharmaceuticals in the Netherlands,^8^ Belgium,^9^ and Germany^10^ demonstrated that discontinuing reimbursement has been problematic. This is also supported by a discrete-choice experiment,^11^ which found that both policy makers and the public find it more difficult to discontinue reimbursement compared with not approving reimbursement in the first place. Our case study highlights that the appropriate type of guidance and a sufficient length of follow-up depends on multiple considerations, such as the decision relevance horizon, the time it takes to reverse an approval decision, the presence of significant irrecoverable costs, the size of the beneficiary population, the technology’s price, and the expected value of the additional data, and is likely to vary across different technology appraisals. For example, AWR was revised to OIR when we assumed a longer delay in reversing an AWR decision. In the scenario in which we assumed a 50% price discount, OIR was revised to approval since the benefits of early patient access given the lower price exceeded the value of additional evidence foregone. Adoption decisions that account for the ENBS of collecting additional survival data can therefore provide incentives for manufacturers to reduce the price when evidence is immature or justify a higher price by investing in better evidence.^13^ While extrapolation of survival is often a key uncertainty, collecting data on other uncertain model parameters such as costs and quality of life may also be valuable, although this may require conducting a new study.

TAs of new oncology drugs, including the case study presented in this tutorial, are routinely informed by PSMs that are constructed using immature survival data. PSMs are relatively easy to construct and interpret, as they use commonly reported clinical endpoints such as OS and PFS directly to estimate health state membership. A key limitation of PSMs is the assumption of independence between survival endpoints.^46^ This is particularly important for OS, which is extrapolated independently of intermediate endpoints such as progression. The independence between survival endpoints limits the possibility of assessing the biological mechanisms that drive the extrapolations, such as the treatment effect on pre- and post-progression mortality. Further research could extend the ENBS calculations for survival data described in this tutorial to the context of state-transition models (STMs), in which patients move between health states at fixed cycle times based on the probability of transitioning from one state to another.^46^ By explicitly modeling the link between intermediate endpoints such as disease progression and survival, STMs can overcome a key limitation of PSMs regarding the independence between survival endpoints. In principle, all the necessary information for applying the methods described in this tutorial can be obtained from a probabilistic sample of transition hazards, since the hazards can be summed up to produce the cumulative hazards, and survival probabilities can be obtained by exponentiating the negative of the cumulative hazards. Survival times can then be simulated by first sampling a PFS time and then deciding whether the sampled PFS time is a progression or death event using a binomial experiment with probability derived from the hazards of transitioning from PFS to PPS and OS.^56^ If the simulated time is a progression event, remaining time until death can be simulated from the conditional survival distribution for PPS to OS. Alternatively, the vectors of OS and PFS probabilities derived from an STM could be used directly as inputs for the ENBS algorithm described in this tutorial, in the same manner as for a PSM. Further research could focus on investigating this approach. A limitation of STMs is that they require IPD to estimate the transition probabilities, which is usually only available to the manufacturer.^46^ Estimating transition probabilities can also be challenging in the context of immature survival data, as there may be only limited available data to inform the transitions, particularly for post-progression mortality. For example, patients who progress early may also have a higher risk of mortality following progression, and since early progressors will initially be overrepresented among the progressed patients, there is a risk of overestimating post-progression mortality. Woods et al.^46^ recommended using STMs alongside PSMs to better assess clinical uncertainties during extrapolation periods. Furthermore, they highlighted the need for further research on methods for estimating transition probabilities in STMs in contexts in which PSMs are used.

Most TAs are informed by cost-effectiveness models that have been developed in Excel, which puts important constraints on the type of analysis that can be performed compared with the use of programming software such as R or Python.^57^ For example, Excel has no option of fitting a generalized additive model or Gaussian process regression, which are required to compute the EVSI using the methods described in this tutorial. It is, however, straightforward to run a PA and store the sampled survival probabilities and net benefits in Excel, which can then be loaded in R as a CSV file and used as input for the EVSI and ENBS functions described in this tutorial.

## Conclusion

This tutorial presents a general-purpose algorithm and practical R code that standardizes ENBS calculations for survival data from traditional probabilistic analyses, such as those used in TAs of new pharmaceuticals.^19–22^ We demonstrated in a case study that our methods are useful not only for supporting the prioritization of new research but also for optimizing reimbursement decisions that are informed by immature survival data from ongoing trials.^55^ We hope this tutorial will contribute to the uptake of value-of-information methods to help health care authorities decide whether current evidence is sufficient for decision making or whether more mature data are needed.

## Supplemental Material

sj-pdf-1-mdm-10.1177_0272989X241279459 – Supplemental material for Calculating the Expected Net Benefit of Sampling for Survival Data: A Tutorial and Case Study Supplemental material, sj-pdf-1-mdm-10.1177_0272989X241279459 for Calculating the Expected Net Benefit of Sampling for Survival Data: A Tutorial and Case Study by Mathyn Vervaart in Medical Decision Making
