# Supplementary material for: Calculating the Expected Net Benefit of Sampling for Survival Data: A Tutorial and Case Study
Source: Med Decis Making. 2024 Sep 20;44(7):719–41. doi: 10.1177/0272989X241279459 (PMC11490075; doi:10.1177/0272989X241279459)
Supplement: sj-pdf-1-mdm-10.1177_0272989X241279459 – Supplemental material for Calculating the Expected Net Benefit of Sampling for Survival Data: A Tutorial and Case Study [file sj-pdf-1-mdm-10.1177_0272989X241279459.pdf]

Calculating the Expected Net Benefit of Sampling for Survival Data: A Tutorial and Case Study. Supplementary online material.

Mathyn Vervaart[1,2,*]

[1]Department of Health Management and Health Economics, University of Oslo, Oslo, Norway
[2]Clinical Trial Unit, Oslo University Hospital, Oslo, Norway

12 August, 2024

*Corresponding author: Mathyn Vervaart, Department of Health Management and Health Economics, University of Oslo, Forskningsveien 3A, Harald Schjelderups hus, 0373 Oslo, Norway (mathyn.vervaart@medisin.uio.no).

Appendices

Appendix A - Reconstructed individual patient data

We reconstructed individual patient data (IPD) for OS and PFS from published Kaplan-Meier (KM) curves.[1] We used the algorithm by Liu et al. (2014)[2] to extract the coordinates of the KM curves from vector images. We derived the event times from the extracted coordinates of the steps in the KM curves. We calibrated the timing of the censoring events to occur before or after a survival event within each 3-month time interval by comparing the resulting survival probabilities with the extracted coordinates of the KM curves, and then distributing the censoring events uniformly between subsequent survival events within each time interval. Details about the `R` code for the reconstruction of the IPD can be found in the accompanying Github repository at https://github.com/matverv/enbs-survival-tutorial.

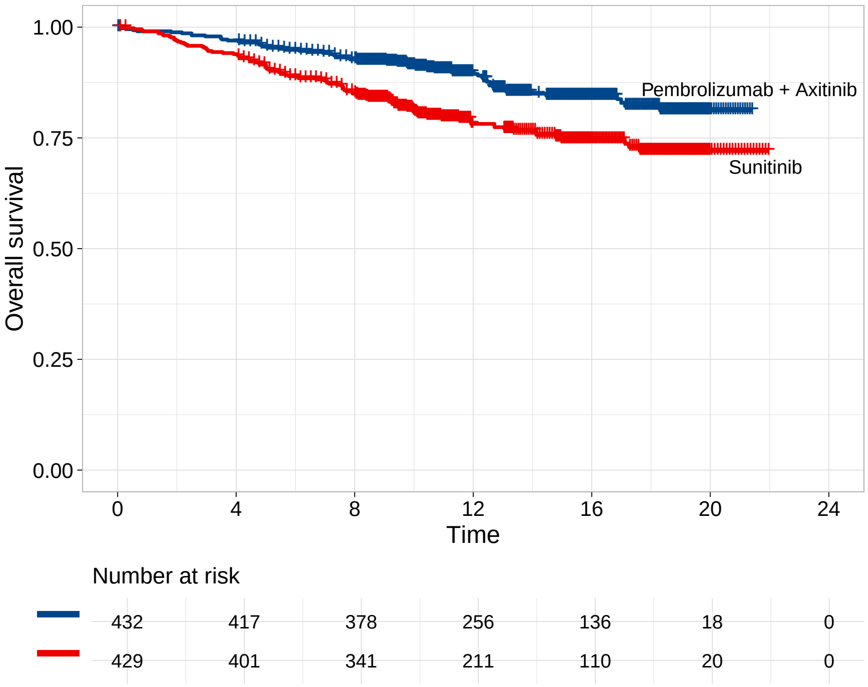

Figure A1: Overall survival for Pembrolizumab plus Axitinib and Sunitinib. Data were reconstructed from the published Kaplan-Meier curves from KEYNOTE-426[1] using the algorithm by Liu et al. (2014)[2].

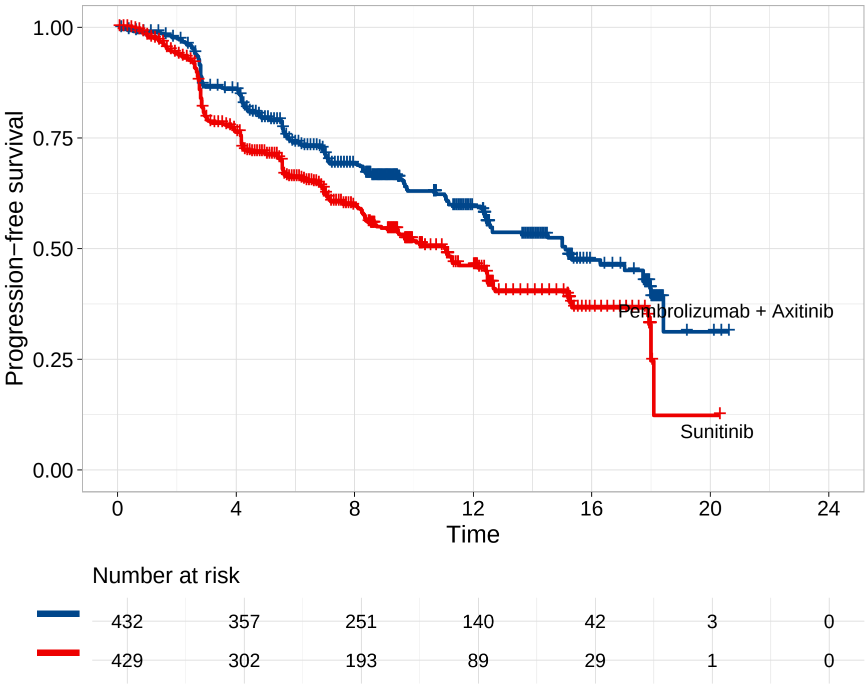

Figure A2: Progression-free survival for Pembrolizumab plus Axitinib and Sunitinib. Data were reconstructed from the published Kaplan-Meier curves from KEYNOTE-426[1] using the algorithm by Liu et al. (2014)[2].

Appendix B - Prior parameter distributions for the case study

Table B1: Prior parameter distributions for the case study

Parameter description	Mean, $\boldsymbol{\mu}$	Covariance matrix, $\boldsymbol{\Sigma}$	Standard error, **SE**	Distribution
**Overall survival, Pembro + Axi**				
Log-logistic log shape<br>Log-logistic log scale	$\begin{pmatrix} 0.19 \\ 5.64 \end{pmatrix}$	$\begin{pmatrix} 0.01 & -0.02 \\ -0.02 & 0.05 \end{pmatrix}$		Bivariate Normal($\boldsymbol{\mu}, \boldsymbol{\Sigma}$)
Exponential log rate	−6.04		0.02	Normal($\boldsymbol{\mu}$, **SE**)
**Overall survival, Suni**				
Exponential log rate	−5.45		0.01	Normal($\boldsymbol{\mu}$, **SE**)
**Progression-free survival[a], Pembro + Axi**				
Exponential log rate	−4.55		0.01	Normal($\boldsymbol{\mu}$, **SE**)
**Progression-free survival[a], Suni**				
Exponential log rate	−4.21		0.00	Normal($\boldsymbol{\mu}$, **SE**)
**Time on treatment, Pembro**				
Weibull log shape<br>Weibull log scale	$\begin{pmatrix} -0.25 \\ 4.62 \end{pmatrix}$	$\begin{pmatrix} 0.06 & -0.00 \\ -0.00 & 0.10 \end{pmatrix}$		Bivariate Normal($\boldsymbol{\mu}, \boldsymbol{\Sigma}$)
**Time on treatment, Axi**				
Exponential log rate	−4.52		0.07	Normal($\boldsymbol{\mu}$, **SE**)
**Time on treatment, Suni**				
Exponential log rate	−4.17		0.06	Normal($\boldsymbol{\mu}$, **SE**)
**Time to death utility parameters**				
0 to 29 days to death	0.46		0.04	Beta(83, 97)
30 to 89 days to death	0.59		0.02	Beta(270, 185)
90 to 179 days to death	0.75		0.02	Beta(389, 130)
180 to 359 days to death	0.77		0.02	Beta(472, 142)
$\geq$ 360 days to death	0.82		0.01	Beta(1194, 255)
Disutility	0.06		0.01	Normal($\boldsymbol{\mu}$, **SE**)
**Drug costs per administration (£)**				
Pembro drug cost[b,c]	789.00		–	Constant
Axi drug cost[b,d]	527.55		–	Constant
Suni drug cost[b,e]	470.82		–	Constant
**Drug dose intensity**				
Pembro dose intensity	0.95		0.05	Normal($\boldsymbol{\mu}$, **SE**)
Axi dose intensity	0.85		0.04	Normal($\boldsymbol{\mu}$, **SE**)
Suni dose intensity	0.75		0.04	Normal($\boldsymbol{\mu}$, **SE**)
**Administration costs (£)**				
Simple parental chemotherapy	174.40		8.89	Gamma(384, 0.5)
Complex chemotherapy	309.20		8.89	Gamma(384, 0.8)
Oral chemotherapy	0		–	Constant

Table B1: Prior parameter distributions for the case study (continued)

Parameter description	Mean, $\boldsymbol{\mu}$	Covariance matrix, $\boldsymbol{\Sigma}$	Standard error, **SE**	Distribution
**Weekly disease management costs (£)**				
PFS state cycle 0	280.05		14.29	Gamma(384, 0.7)
PFS state subs. cycles	51.05		2.60	Gamma(384, 0.1)
Progressed state	51.05		2.60	Gamma(384, 0.1)
**Subsequent treatment costs (£)**				
Subs. treatment Pembro + Axi	$19,096.77$		$1,909.67$	Gamma(100, 191.0)
Subs. treatment Suni	$24,700.62$		$2,470.06$	Gamma(100, 247.0)
Terminal care	$8,073$		411.90	Gamma(384, 21.0)
**Adverse event costs (£)**				
Adverse events Pembro + Axi	379.90		19.38	Gamma(384, 1.0)
Adverse events Suni	379.90		19.38	Gamma(384, 1.0)
**Other parameters**				
Annual discount rate	0.035		–	Constant
Monetary value (£) of 1 QALY	$30,000$		–	Constant

[a] Kaplan-Meier data is used up until month 12.
[b] Assumed value-based price.
[c] Administered every 3rd week.
[d] Administered every 4th week.
[e] Administered every 6th week.

Data sources

Survival model parameters

We fitted survival models to the IPD reconstructed for OS and PFS (detailed in Appendix A) using the `flexsurv` package in `R`[3], obtaining the maximum likelihood estimates of the model parameters. Time on treatment model parameters were sourced from the company submission as reported in TA650.[4]

Utility values

We obtained the values for the time to death utility parameters from the study by Bensimon et al. (2020)[5].

Dose intensity

We obtained the dose intensities for Pembrolizumab, Axitinib and Sunitinib from technology appraisal ID2019_045 by the Norwegian Medicines Agency[6].

All other parameter values were derived from the committee papers in TA650[4].

Appendix C - Validation of the replicated health economic model

Table C1: Discounted results for the company's base case as reported in TA650[4].

Technologies	Costs	LYs	QALYs	Incremental costs	Incremental QALYs	ICER
Sunitinib	NR	3.864	NR	-	-	-
Pembrolizumab + Axitinib	NR	6.887	NR	£137,537	2.320	£59,292

LY, life year; QALY, quality-adjusted life year; ICER, Incremental cost-effectiveness ratio (cost per QALY gained); NR, not reported.

Table C2: Discounted results for the reconstructed company's base case analysis.

Technologies	Costs	LYs	QALYs	Incremental costs	Incremental QALYs	ICER
Sunitinib	£67,187	3.874	5.322	-	-	-
Pembrolizumab + Axitinib	£207,112	6.938	2.975	£139,926	2.347	£59,624

LY, life year; QALY, quality-adjusted life year; ICER, Incremental cost-effectiveness ratio (cost per QALY gained).

Appendix D - Probabilistic analysis output

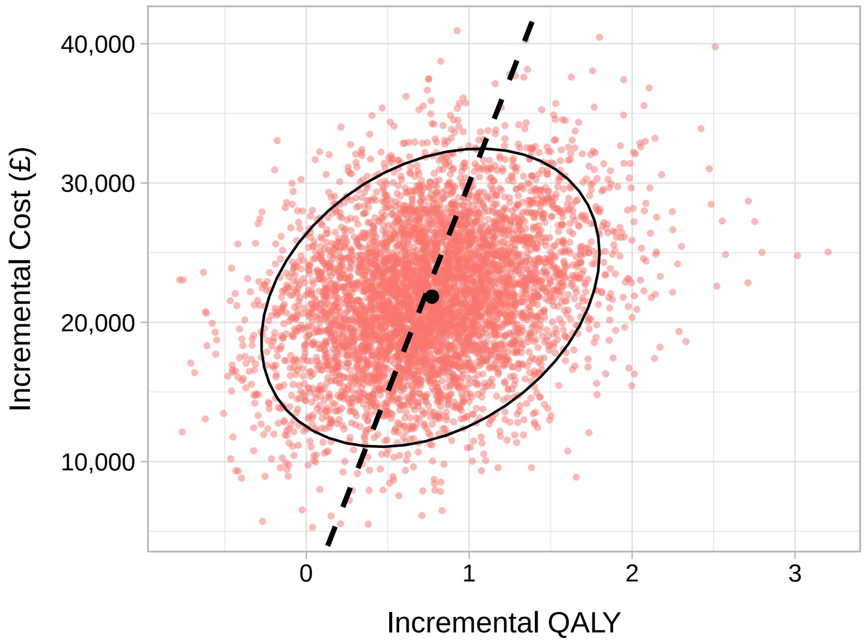

Figure D1: Cost-effectiveness scatterplot for Pembrolizumab plus Axitinib compared to Sunitinib.

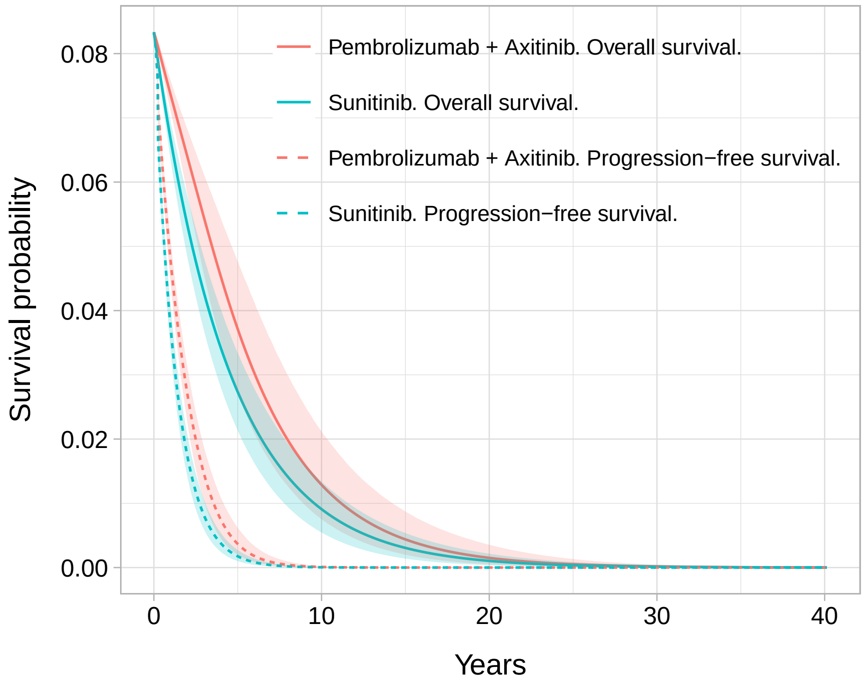

Figure D2: Expected survival curves and 95% confidence bounds for overall survival and progression-free survival for Pembrolizumab plus Axitinib and Sunitinib with adjustment for treatment effect waning.

References

1. Rini BI, Plimack ER, Stus V, et al. Pembrolizumab plus Axitinib versus Sunitinib for Advanced Renal-Cell Carcinoma. *New England Journal of Medicine* 2019; 380: 1116–1127.

2. Liu Z, Rich B, Hanley JA. Recovering the raw data behind a non-parametric survival curve. *Systematic Reviews*; 3. Epub ahead of print December 2014. DOI: 10.1186/2046-4053-3-151.

3. Jackson C. Flexsurv: A Platform for Parametric Survival Modeling in R. *Journal of Statistical Software* 2016; 70: 1–33.

4. National Institute for Health and Care Excellence. Pembrolizumab with axitinib for untreated advanced renal cell carcinoma [TA650].

5. Bensimon AG, Zhong Y, Swami U, et al. Cost-effectiveness of pembrolizumab with axitinib as first-line treatment for advanced renal cell carcinoma. *Current Medical Research and Opinion* 2020; 36: 1507–1517.

6. Norwegian Medicines Agency. Pembrolizumab with axitinib for untreated advanced renal cell carcinoma (ID2019_045).
